# Health-related quality of life in people with type 1 Diabetes Mellitus: data from the Brazilian Type 1 Diabetes Study Group

**DOI:** 10.1186/s12955-015-0396-0

**Published:** 2015-12-24

**Authors:** Ana Carolina Contente Braga de Souza, João Soares Felício, Camila Cavalcante Koury, João Felício Abrahão Neto, Karem Barbosa Miléo, Flávia Marques Santos, Carlos Antonio Negrato, Ana Regina Bastos Motta, Denisson Dias Silva, Thaís Pontes Arbage, Carolina Tavares Carvalho, Hana Andrade de Rider Brito, Elizabeth Sumi Yamada, Franciane Trindade Cunha de Melo, Fabricio de Souza Resende, Juliana Cristina Cardoso Ferreira, Marilia Brito Gomes, On behalf of the Brazilian Type 1 Diabetes Study Group (BrazDiab1SG)

**Affiliations:** University Hospital João de Barros Barreto, Federal University of Pará, Endocrinology Division, Mundurucus Street, 4487, Guamá, 66073-000 Belém, Pará Brazil; Bauru’s Diabetics Association, Internal Medicine, Nações Unidas Avenue, 28040, Centro, 17010-130 Bauru, São Paulo Brazil; State University of Rio de Janeiro, Diabetes Unit, Boulevard 28 de setembro, 77, Vila Isabel, 20551-030 Rio de Janeiro, Rio de Janeiro Brazil

**Keywords:** Type 1 Diabetes Mellitus, Health-related quality of life, EuroQol

## Abstract

**Background:**

Type 1 Diabetes Mellitus (Type 1 DM) affects the psychological and emotional well-being of patients and their families. This study aims to evaluate the health- related quality of life (HRQoL) of people with Type 1 DM in Brazil, a country of continental proportions, using the EuroQol questionnaires.

**Methods:**

This was a retrospective, cross-sectional, multicenter study performed by the *Brazilian Type 1 Diabetes Study Group*, by analyzing EuroQol scores from 3,005 participants with Type 1 DM, in 28 public clinics in Brazil. Data on demography, economical status, chronic complications, glycemic control and lipid profile were also collected.

**Results:**

The assessment of HRQoL by the EuroQol showed that the average score assigned to general health in Brazil is markedly lower than those found in two other Type 1 DM population-based studies conducted in Europe (EQ–VAS from the Netherlands, the United Kingdom and Brazil were 80.8 ± 15.2, 75.1 ± 18.4 and 72.5 ± 22, respectively). Additionally, our data suggest that a better glycemic control could positively impact the HRQoL of people with Type 1 DM, implying that each 1 % reduction in glycated haemoglobin might lead to an increase of 1.5 points in general health status assessed by the EuroQol.

**Conclusions:**

This is a population-based study evaluating the HRQoL of people with Type 1 DM in Brazil. Our data indicate a worse quality of health of people with Type 1 DM in Brazil in comparison to Europe, and suggest that a better glycemic control could positively impact the HRQoL of these individuals. However, this study points to the existence of additional factors not yet evaluated that could be determinant in the HRQoL of these people.

## Background

Type 1 Diabetes Mellitus (Type 1 DM) affects the psychological and emotional well-being of patients and their families [1]. It has been shown that individuals with diabetes have reduced health-related quality of life (HRQoL) compared with the general population [2], and that poor HRQoL in patients with diabetes is associated with adverse outcomes, including increased mortality [3]. However, most of the studies on diabetes and HRQoL have been conducted in type 2 diabetic patients, and data in developing countries are still limited [4–6].

A large number of factors might be responsible for the negative impact of diabetes on quality of life (QoL), such as fear of hypoglycemia or of secondary complications, future worries on stigmatization, loss of flexibility, poor self-image, overprotection, age, economic class, education, ethnicity, knowledge regarding the disease and the daily demands of diabetes therapy (i. e. insulin therapy, blood glucose monitoring) [7–9]. The impact of glycemic control on the QoL of people with diabetes remains controversial. Some studies have shown no effect of an intensive glycemic control on QoL [10, 11]. In opposition, the *Hvidoere Study Group* has suggested that the outcomes of diabetes care, assessed by glycated haemoglobin (HbA1c), are better in individuals with a better QoL [12].

When addressing this topic, in order to avoid using confusing terminology, which could lead to misleading conclusions, it is important to clarify that health status, quality of health, HRQoL, and QoL cannot be assumed as interchangeable terms [13]. QoL is a multifaceted and dynamic concept, considering its highly subjective and individual nature [14, 15]. It has been defined as “how good or bad a person feels their life to be” [15]. Health status, quality of health and HRQoL, on the other hand, refer to how people feel about their health (physical and mental) [4]. Therefore, quality of health and QoL are not the same thing [15]. Impaired health or well-being may lead to, or be experienced at the same time as impaired quality of life—but not necessarily. Furthermore, excellent health does not infer excellent quality of life [16].

Once established the conceptual difference between these terms, it is also essential to determine the instruments suitable to assess each of them in diabetes research. A systematic literature search, including over 6,000 abstracts, found that some instruments are better to assess QoL [World Health Organization Quality of Life (WHOQOL), Diabetes Quality of Life (DQOL) and Audit of Diabetes-Dependent Quality of Life (ADDQoL)], and others more accurately measure health status [Short-Form 36 (SF-36), EuroQoL 5-Dimension (EQ-5D)] [14].

The Brazilian Type 1 Diabetes Study Group performed a survey that analyzed the demographic, clinical, laboratorial and economic data of 3,591 patients with Type 1 DM who received medical care at public clinics in Brazil. The purpose of the present study was to analyze the association between a better glycemic control and HRQoL, by using the EuroQol questionnaires, as well as investigate additional variables that could influence the HRQoL of these individuals. In parallel, we aimed to evaluate the HRQoL of people with Type 1 DM in the Southern hemisphere, in a country of continental proportions such as Brazil, and establish a comparison with worldwide data.

## Methods

### Study design

The *Brazilian Type 1 Diabetes Study Group* performed a retrospective, cross-sectional and multicenter study on people with Type 1 DM between December 2008 and December 2010, in 28 public clinics of the secondary and tertiary care level, located in 20 cities (population greater than 100,000), in the five Brazilian geographical regions (North, Northeast, Southeast, South and Midwest). For statistical purposes, North and Northeast were grouped.

### Patients

All patients received health care from the National Brazilian Health Care System (NBHCS), and were treated by an endocrinologist in secondary or tertiary care settings. Although the study did not include people treated at primary level, these individuals represent a minority of people with Type 1 DM in Brazil. We included on this study patients diagnosed with Type 1 DM by a physician (based on a typical clinical presentation as well as the need of using insulin continuously since the diagnosis), with follow-up time in each center greater than or equal to 6 months, and older than 10 years old. Exclusion criteria were pregnancy, lactation, history of acute infectious processes or diabetic ketoacidosis in the 3 months prior to assessment.

### Clinical and laboratorial data

Data of interest for the analysis of HRQoL were obtained from questionnaires self-completed by the patients, in addition to records obtained from medical charts. Trained physicians interviewed and examined all patients according to a standardized protocol, assessing current age, age at diagnosis of Type 1 DM, duration of diabetes (years), monthly family income in minimum wages, economic class, physical activity (times per week) [17], height (m), weight (kg), blood pressure (mmHg), insulin therapy regimen, diabetes-related comorbidities, frequency of daily self-monitoring of capillary blood glucose and smoking status (defined as smoking more than one cigarette per day at the time of the interview) [18].

The economic status was defined according to the Brazilian Economic Classification Criteria, which also considers education level, categorized as illiterate/incomplete primary education, complete primary education/incomplete secondary education, complete secondary education/incomplete high school, complete high school/some college, or complete college education. For this analysis, the economic classes considered were high, medium, low and very low [19].

Body mass index (BMI) (Kg/m^2^) was determined by dividing the weight (kg) by height (m) squared. In adults (people older than 19 years old) [20], overweight was defined as a BMI ≥25 kg/m^2^ and obesity as a BMI ≥30 kg/m^2^ [21]. In children (people younger than 13 years old) and adolescents (people between 13 and 19 years old) [20], overweight was defined as a BMI ≥85th percentile for age and gender, and obesity as a BMI ≥95th percentile for age and gender [22].

The levels of HbA1c, fasting plasma glucose (FPG), total cholesterol, LDL cholesterol, HDL cholesterol, and triglycerides measured during the last clinical visit were obtained from the participants’ medical records. Within one year of the study assessment, people with a diabetes duration greater than or equal to five years from diagnosis were screened for the following chronic diabetes-related complications: retinopathy, clinical nephropathy, macrovascular diseases (classified as clinical coronary artery disease, stroke, and peripheral vascular disease) and foot pathologies.

HbA1c values were obtained using high-performance liquid chromatography (HPLC) in 54.6 % and turbidimetry and in 40 % of cases; in the remaining patients we used other methods. Levels of fasting and postprandial glycemia, total cholesterol, HDL cholesterol and triglycerides were measured by enzymatic techniques. LDL cholesterol level was calculated using the Friedewald’s equation. The American Diabetes Association’s (ADA) goals for adequate metabolic and clinical control were adopted by the Brazilian Type 1 Diabetes Study Group.

### Health-related quality of life assessment

HRQoL was assessed by the EuroQol [23], a measure of health status that includes two tools: the EQ-5D and the EQ-VAS. The first one analyzes descriptively five dimensions of problems (mobility, self-care, usual activities, pain and discomfort, and anxiety and depression) on a scale of three scores, graded from 1 to 3 (1 “I have no problems”, 2 “I have some problems, ”3“ I have extreme problems”). The EQ-VAS (overall health status) consists of an analog scale from 0 (very bad state of health) to 100 (optimal health status), for the patient to check or tell a value that reflects their perception of state of health. The EuroQol is not reliable in individuals younger than 10 years old. Therefore, these participants were excluded from the initial sample.

Data collected using EQ-5D can be presented in various ways: presenting results from the descriptive system as a health profile, presenting results of the EQ- VAS as a measure of overall self-rated health status, and presenting results from the EQ-5D index value. In order to obtain the EQ-5D index value, it is required a general population-based value set (as opposed to a patient-based set). The rationale behind this is that the values are supposed to reflect the preferences of local taxpayers and potential receivers of healthcare. However, in Brazil, there is not a population-based index available yet. Information in the single index format is useful, for example, in cost utility analysis, which is not the purpose of the present study.

### Statistical analysis

The study sample represented the distribution of Type 1 DM cases across four geographical regions in Brazil, estimated using the overall population distribution reported in the Brazilian Institute of Geography and Statistics’ (IBGE) population census of 2000 [24]. These data were combined with the national estimates of diabetes prevalence, which were derived from a 1988 survey, to determine the minimum number of patients to be studied in each region [25]. Three thousand four hundred fifty seven patients would be necessary to identify a proportion of patients with adequate glycemic control of 10 %, with a 95 % confidence and a margin of error of 1 %. This number was rounded to 3,500 patients and distributed among the four geographical regions [North: 266 (7.6 %); Northeast: 984 (28.1 %); Southeast: 1492 (42.6 %); South: 518 (14.8 %); and Midwest: 240 (6.9 %) individuals]. A total of 3,591 patients were included in this study. Participants younger than 10 years old were excluded from the initial sample of 3,591 individuals, so that the final sample comprised 3,005 patients.

Categorical variables were presented as frequency (percentage). All normally distributed values were given as mean ± standard deviation (SD) and all other values were given as median (range). We used Chi-squared and Fisher tests to compare categorical data, and Student-T and Man-Whitney tests for comparisons between two groups with numeric variables. To test the differences among more than two groups, analysis of variance (ANOVA) was performed for all normally distributed variables, and Kruskal-Wallis test was used for the non-normally distributed variables. For multiple comparisons, the Tukey test (post-hoc) was used. For correlation analysis, Pearson or Spearman tests were used.

A model of simple linear regression was used with the EQ-VAS score as dependent variable and HbA1c as independent variable. In addition, we performed a model of multiple regression analysis (stepwise forward), with EQ-VAS score as dependent variable and HbA1c, practice of physical activity, duration of diabetes, age, and micro and macrovascular complications as independent variables. We have not included in the model the variables that did not present strong correlation with EQ-VAS (monthly family income and economic status), and FPG, which is inferior in reflecting glycemic control in type 1 diabetic patients when compared to HbA1c. For statistical purposes (both comparison and regression models), we used the HbA1c levels measured by HPLC.

A two-sided *p* < 0.05 was considered statistically significant. All data was stored and processed by EPIINFO 2000 and analyzed using the Statistical Package for the Social Sciences version 20.0 (IBM, Chicago, IL, USA) and Sigma Stat version 3.5 (Jandel Scientific Corporation, Chicago, IL, USA).

### Ethics, consent and permissions

Each center’s local ethics committee approved the study (Appendix 1). Written informed consent was obtained from all of the patients, or their legal parents or guardians for children.

## Results

The general demographic and clinical data of the assessed population are shown in Table 1.

**Table 1:** General demographic and clinical data of people with Type 1 DM in Brazil

Variables	
Participants, n	3,005
Age, years ± SD	23.9 ± 10.8
Women, n (%)	1,700 (56)
Age at diagnosis of Type 1 DM in years, n (%)	
0–4.9	330 (11)
5–9.9	721 (24)
10–14.9	932 (31)
15–19.9	492 (16.4)
20–29.9	414 (13.5)
≥ 30	126 (4.1)
Mean age at diagnosis of Type 1 DM, years ± SD	13 ± 7.9
Duration of Type 1 DM, years	10.9 (7 months – 50 years)
Ethnicity, n (%)	
Caucasian	1,720 (57.2)
Non-Caucasian	1,285 (42.8)
Economic class, %	
High	7.7
Medium	24.3
Low	34.2
Very low	66.1
Geographic Region, n (%)	
Southeast	1,180 (39.3)
North-Northeast	925 (30.8)
South	718 (23.9)
Midwest	182 (6.0)
Monthly family income, number of minimum wages ± SD	2.3 ± 1.4
Practice of physical activity, times per week ± SD	3.6 ± 1.4
Microvascular complications, n (%)	673 (29.6)
Macrovascular complications, n (%)	124 (5.5)
Type 1 DM treatment, %	
Intermediate-acting insulin	16.3
Long-acting insulin	1.3
Insulin pump	1.5
Intermediate- or long- and short-acting insulin	80.8
Amount of insulin usage, UI/Kg/day ± SD	0.9 ± 0.4

Clinical and laboratorial data and the percentage of participants who met the ADA’s criteria for appropriate metabolic and clinical control are shown in Table 2. Overweight was observed in 586 (19.5 %) participants. Screening for chronic complications had not been performed on the year prior to the study in 735 people (24.4 %). Glycemic control was considered poor in 2,571 (85 %) individuals. The description of the EuroQol results in the population of people with Type 1 DM in Brazil also is presented in Table 2.

**Table 2:** Clinical and laboratorial data and participants at ADA’s goal for adequate metabolic and clinical control and evaluation of HRQoL of people with Type 1 DM in Brazil by EuroQol

Variables	Mean ± SD	Participants at goal N (%)
BMI, Kg/m^2^	22.6 ± 3.9	2,077 (70)
HbA1c, % (mmol/mol)	9.4 ± 2.4 (79 ± 3)	434 (15)
Fasting plasma glucose, mmol/l	10.2 ± 5.8	765 (28)
Total cholesterol, mmol/l	4.4 ± 1.1	1,854 (73)
Triglycerides, mmol/l	1.1 ± 0.8	1,919 (78)
LDL cholesterol, mmol/l	2.6 ± 2.1	1,403 (59)
HDL cholesterol, mmol/l	1.4 ± 0.4	1,809 (75)
EQ-VAS	72.5 ± 22	-
Pain and discomfort	1.37 ± 0.5	-
Anxiety and depression	1.63 ± 0.6	-
Usual activities	1.1 ± 0.3	-
Self-care	1.03 ± 0.2	-
Mobility	1.07 ± 0.3	-

The assessment of health status by the EQ-VAS shows that the average score assigned to general health in Brazil is markedly lower than those found in two other Type 1 DM population-based studies conducted in the Northern hemisphere, as demonstrated in Table 3. Our results indicated an average HbA1c of 9.4 % (79 mmol/mol), higher than the mean values found in the Netherlands (8.3 % or 67 mmol/mol) [26] and in the United Kingdom (8.2 % or 66 mmol/mol) [27].

**Table 3:** EQ- VAS scores in population-based studies on HRQoL in Type 1 DM

Site	Participants (N)	EQ-VAS	Authors (Year)
Netherlands	274	80.8 ± 15.2	Hart et al. (2003) [23]
United Kingdom	459	75.1 ± 18.4	Hopkins et al. (2012) [27]
Brazil	3,005	72.5 ± 22	Souza et al. (2015)

The Table 4 show the correlation between EQ-5D and the variables that could possibly affect HRQoL.

**Table 4:** Correlation between EQ-5D and variables that could possibly affect HRQoL

Variables		Pain and discomfort	Anxiety and depression	Usual activities	Self-care	Mobility
Age (years)	r	0,23	0,06	0,22	0,12	0,21
	p	<0.05	<0.05	<0.05	<0.05	<0.05
Duration of diabetes (years)	r	0,15	0,04	0,17	0,11	0,18
	p	<0.05	<0.05	<0.05	<0.05	<0.05
HbA1c % (mmol/mol)	r	0.05	0,05	−0,02	−0,01	−0,03
	p	<0.05	<0.05	NS	NS	NS
Micro and macrovascular complications (%)	r	−0,18	−0,07	−0,18	−0,09	−0,20
	p	<0.05	<0.05	<0.05	<0.05	<0.05
Physical activity (times per week)	r	−0,10	−0,07	−0,09	−0,08	−0,09
	p	<0.05	<0.05	<0.05	<0.05	<0.05

*NS* not significant

Furthermore, we evaluated the clinical and laboratorial variables that could influence the HRQoL of people with Type 1 DM and the existence of possible correlations. Individually, age, duration of diabetes, fasting blood glucose and the presence of chronic complications presented very poor correlations with EQ-VAS. The most significant correlation was between EQ-VAS and HbA1c (Table 5). Evaluating the simple linear regression model, using the EQ-VAS score as a dependent variable and HbA1c as independent variable, we found r^2^ = 0.04, p <0.001, with an unstandardized coefficient (Beta 1) equal to −1.5 (95 % CI = −1.772 to −1.197). It implies that a 1 % reduction of HbA1c may lead to a 1.5 point elevation of overall health status.

**Table 5:** Correlation between EQ-VAS and the variables that could possibly affect HRQoL

Variables	EQ-VAS	
	r	P
Age (years)	−0.1	<0.05
Duration of diabetes (years)	−0.1	<0.05
Practice of physical activity (times per week)	0.15	<0.05
Economic status %	−0.05	<0.05
Monthly family income number of minimum wages	0.03	NS
Fasting plasma glucose mmol/l	−0.1	<0.05
HbA1c % (mmol/mol)	−0.2	<0.05
Microvascular complications (%)	−0.1	<0.05
Macrovascular complications (%)	−0.1	<0.05
Micro and macrovascular complications (%)	−0.1	<0.05

*NS* not signifficant

Finally, in the multiple regression model (stepwise forward), with EQ-VAS score as dependent variable and HbA1c, practice of physical activity, duration of diabetes, age and micro and macrovascular complications as independent variables, all of them were included on the model, with *p* <0.05. Even though HbA1c was the most important of the model, all variables combined were capable of determining only 7.1 % of the overall health status of people with Type 1 DM.

On a post-hoc analysis, we found that the North-Northeast region presents a higher index in the assessment of the overall health status compared to the Southeast (North-Northeast = 74.6 ± 30 and Southeast = 70.4 ± 19, p <0.05). Additionally, the EQ-5D showed a markedly lower frequency of self-reported anxiety-depression in the North-Northeast compared to the other regions of the country (North-Northeast = 1.53 ± 0.6, Southeast = 1.65 ± 0.7, South = 1.72 ± 0.7 and Midwest = 1.67 ± 0.7, p <0.05).

## Discussion

This is a population-based study evaluating the HRQoL of people with Type 1 DM in the Southern hemisphere, in a country of continental proportions such as Brazil. The assessment of HRQoL by the EuroQol showed that the average score assigned to general health in Brazil is markedly lower than those found in two other Type 1 DM population-based studies conducted in Europe. Additionally, our data suggest that a better glycemic control could positively impact the health status of people with Type 1 DM.

Since no Brazilian national data on the HRQoL of people with Type 1 DM were available, we compared the overall health status found in this study with those determined by similar European researches. Brazilian participants reported an inferior HRQoL when compared to individuals with Type 1 DM in the Netherlands [26] and in the United Kingdom [27]. The significant difference between the EQ-VAS scores found in the Northern and Southern hemispheres may be related to the better socioeconomic status, health care system, level of education and glycemic control of the European population. In the present study, the majority of the 3,005 participants evaluated presented poor glycemic control, overweight and were not systematically screened for clinical complications of diabetes. These findings can be considered alarming and suggest that it is necessary and urgent to improve the health care of people with Type 1 DM in Brazil.

The effect of glycemic control on HRQoL of people with Type 1 DM remains controversial, with conflicting studies pointing to opposite directions. The *Diabetes Control and Complications Trial* (DCCT) [10] showed that people with Type 1 DM undergoing intensive diabetes treatment do not face changes in the quality of their lives, even while the rigor of their diabetes care was increased. Additionally, HbA1c levels were not associated with HRQoL or any other psychosocial factors in a cohort of 52 adolescents with Type 1 DM. This study suggested that even teenagers who are successfully achieving HbA1c goals of therapy may perceive diabetes as having a negative impact on their lives, be depressed, and find diabetes difficult to manage [11]. In opposition, Hoey et al. [28], evaluating over 2,000 adolescents with Type 1 DM, found that better HbA1c levels were associated with lower impact, fewer worries, greater satisfaction, and better health perception. Furthermore, the *Hvidoere Study Group* has suggested that the outcomes of diabetes care, assessed by HbA1c, are better in people with a better QoL [12]. In addition, Hopkins et al. [27] demonstrated that the U.K.-based Dose Adjustment for Normal Eating (DAFNE), a structured education program delivered in routine clinical practice, not only improves HbA1c but also reduces psychological distress (anxiety and depression) and improves perceived well-being assessed by the EuroQol in Type 1 DM. Finally, Cochran et al. [29], in a meta-analysis including 1,892 subjects with type 1 or 2 diabetes, documented that people with diabetes experience improved QoL from participation in diabetes self-management training programs, which is associated with better metabolic control. Our results suggest that a better glycemic control could be a tool for enhancing health status, but further prospective studies with intervention are necessary to confirm this hypothesis.

In our study, variables such as age, economic class, physical activities, micro and macrovascular complications, diabetes duration and fasting blood glucose individually showed poor correlation with health status. Hart et al. [30] also showed weak correlations between HRQoL and micro and macrovascular complications. It suggests the existence of additional variables not yet evaluated that could be determinant of HRQoL in those individuals. It has been reported that diabetes mellitus is associated with lower levels of vitamin D [31], which might increase the prevalence of numerous comorbidities, such as depression [32]. In our study, the higher EQ-VAS score and lower frequency of self-reported anxiety-depression in the North-Northeast region of Brazil could be influenced by vitamin D deficiency. Decreased vitamin D levels tend to be more common in regions of lower sunlight exposure, such as the South and Southeast of Brazil. In a study of 102 non-institutionalized and low-income elderly, in Porto Alegre (Brazilian South region), the prevalence of vitamin D deficiency reached 85.7 % [33]. On the other hand, a study in children in Recife (Brazilian Northeast region) virtually detected no cases of vitamin D deficiency [34]. Other factor possibly related to a poorer quality of health could be the stressful lifestyle of largely populated cities. The lower frequency of anxiety-depression found in Brazilian North-Northeast, a region with smaller population density, reinforces that hypothesis.

The main strengths of this study are as follows: 1) the development a population-based study on HRQoL of people with Type 1 DM in the Southern hemisphere; 2) the recruitment of a large and representative population-based cohort with Type 1 DM, in a country of continental proportions such as Brazil; 3) the construction of a national database for tracking these individuals in the long term.

It is also pertinent to reflect on potential shortcomings. The present study may be limited by the self-report nature of doctor-diagnosed diabetes, as there could be misclassification of diabetes, once C-peptide and autoantibodies were not measured. Moreover, all participants of this study were from secondary and tertiary levels of care, and lived in large cities. Although the information about the HRQoL of people treated at primary level has not been achieved, these individuals probably represent a minority of people with Type 1 DM in Brazil. Additionally, the lack of standardization for the assessment of HbA1c could have influenced the results, but in most cases both methods were used and a strong correlation was observed between them. Furthermore, the lack of standard measures and scales makes it difficult to compare the results of different populations on HRQoL [35]. However, proving to be an extremely useful and reliable tool, the EuroQol questionnaire has been used in most global multicenter studies evaluating health status in patients with diabetes. In Brazil, there is not a validated EQ-5D index yet, which would allow us to compare the data more precisely. Thus, the present study is a pilot that must be expanded. Additionally, we did not dispose of the individual data from the European studies to compare to ours. It would also be of great interest to perform a meta-analysis including worldwide trials on HRQoL in people with Type 1 DM, so we could determine more precisely the variables involved in the overall health status, and develop strategies to improve the well-being and satisfaction of these individuals.

## Conclusions

In conclusion, this is a population-based study evaluating the HRQoL of people with Type 1 DM in the Southern hemisphere and in a country like Brazil, of continental proportions. Our data indicate a worse quality of health of people with Type 1 DM in Brazil in comparison to Europe, and suggest that a better glycemic control could positively impact the HRQoL of these individuals. However, our study points to the existence of additional factors not yet evaluated that could be determinant in the HRQoL of people with Type 1 DM. A meta-analysis including worldwide trials would be very useful to identify these possible factors.

## Appendix 1

### Brazilian Type 1 Diabetes Study Group (BrazDiab1SG)

Executive steering committee: Marilia Brito Gomes (chair), Roberta Cobas, Sergio Atala Dib, Carlos Antonio Negrato.

Principal investigators are indicated by an asterisk. Program coordinators are in italics.

Department of Internal Medicine, Diabetes Unit, State University of Rio de Janeiro, Brazil: Roberta Cobas*, M.D. (robertacobas@gmail.com), *Alessandra Matheus*, M.D. (alessandramatheus79@yahoo.com), Lucianne Tannus, M.D. (luciannetannus@ig.com.br), Catia Cristina Sousa Palma, M.D. (catiasousapalma@gmail.com), Leticia Japiassu, M.D. (leticiamauricio@gmail.com), Marilia Brito Gomes, M.D. (mariliabgomes@gmail.com), João Regis Ivar Carneiro, M.D. (endoregis@uol.com.br); Federal University Hospital of Rio de Janeiro: Melanie Rodacki*, M.D. (mrodacki2001@yahoo.com.br), *Lenita Zajdenverg*, M.D. (lenitazaj@gmail.com); General Hospital of Bonsucesso: Neuza Braga Campos de Araújo*, M.D. (russarj@terra.com.br), *Marilena de Menezes Cordeiro*, M.D. (marmecor@gmail.com); University Hospital Clementino Fraga Filho – Children Institute Martagão Teixeira: Jorge Luiz Luescher*, M.D. (luescher_@hotmail.com), *Renata Szundy Berardo*, M.D. (rszundy@br.inter.net); Diabetes Unit, University Hospital of São Paulo, São Paulo: Marcia Nery*, M.D. (marcianery@hcnet.usp.br), *Catarina Cani*, M.D. (catarinagcani@gmail.com), Maria do Carmo Arruda Marques, M.D. (mcarruda@hcnet.usp.br); Pediatric Unit of Endocrinology - Santa Casa Hospital, São Paulo: Luiz Eduardo Calliari*, M.D. (calliari.cidep@uol.com.br), *Renata Maria de Noronha*, M.D. (renata_noronha@uol.com.br); Children Institute of Endocrinology, University Hospital of São Paulo, São Paulo: Thais Della Manna*, M. D. (thais.manna@icr.usp.br), *Roberta Savoldelli*, M.D. (robysds@hotmail.com), Fernanda Garcia Penha, M.D. (fepenha@ajato.com.br); Ribeirão Preto Medical School of São Paulo University, Sao Paulo: Milton Cesar Foss*, M.D. (mcfoss@fmrp.usp.br), *Maria Cristina Foss-Freitas*, M.D. (crisfoss@fmrp.usp.br); Department of Internal Medicine, Medical School, State University of São José do Rio Preto: Antonio Carlos Pires*, M.D. (fpires@terra.com.br), *Fernando Cesar Robles*, M.D. (roblesmed@ig.com.br); Bauru’s Diabetics Association, Bauru, São Paulo: Carlos Antonio Negrato*, M.D. (carlosnegrato@uol.com.br), *Maria de Fatima Guedes*, M.D. (tatiguedeses@hotmail.com); Diabetes Unit, Federal University of São Paulo State, São Paulo: Sergio Atala Dib*, M.D. (sergio.dib@unifesp.br), *Patricia Dualib*, M.D. (patricia.dualib@uol.com.br); Endocrinology Unit, Hospital of Santa Casa of Belo Horizonte, Minas Gerais: Saulo Cavalcanti da Silva*, M.D. (scsendocrino@yahoo.com.br), *Janice Sepúlveda*, M.D. (janicesepulveda@terra.com.br); Diabetes Unit, State University Hospital of Londrina, Paraná: *Emerson Sampaio*, M.D. (emersamp@hotmail.com); Clinical Hospital of the Federal University of Paraná: Rosangela Roginski Rea*, M.D. (rosangelarea@uol.com.br), *Ana Cristina Ravazzani de Almeida Faria*, M.D. (aravazzani@uol.com.br); Institute of Diabetic Children, Rio Grande Sul: Balduino Tschiedel*, M.D. (badutsch@gmail.com), *Suzana Lavigne*, M.D. (suzanalavigne@yahoo.com.br), Gustavo Adolfo Cardozo, M.D. (byguga@hotmail.com); Clinical Hospital of Porto Alegre, Rio Grande do Sul: Mirela Azevedo*, M.D. (mirelajobimazevedo@gmail.com), *Luis Henrique Canani*, M.D. (luishenriquecanani@gmail.com), Alessandra Teixeira Zucatti, M.D. (alezucatti@hotmail.com); University Hospital of Santa Catarina: Marisa Helena Cesar Coral*, M.D. (marisahcc@uol.com.br), *Daniela Aline Pereira*, M.D. (danialine@yahoo.com); Endocrinology and Diabetes Institute of Joinville, Santa Catarina: Luiz Antonio de Araujo*, M.D. (luiz@endoville.com.br); Regional Hospital of Taguatinga, Brasília: Hermelinda Cordeiro Pedrosa*, M.D (pedrosa.hc@globo.com), *Monica Tolentino*, M.D. (monicatolentino@uol.com.br), Flaviene Alves Prado, M.D. (draflavienne@gmail.com); General Hospital of Goiânia: Nelson Rassi*, M.D. (nrassi@brturbo.com.br), *Leticia Bretones de Araujo*, M.D. (leticiabretones@yahoo.com.br); Diabetes and Endocrinology Center of Bahia: Reine Marie Chaves Fonseca*, M.D. (reinemar@terra.com.br); *Alexis Dourado Guedes*, M.D. (dr.alexis@uol.com.br), Odelisa Silva de Mattos, M.D. (odelisam@yahoo.com.br); Federal University of Maranhão: Manuel Faria*, M.D. (mfaria@inlab.com.br), *Rossana Azulay*, M.D. (rossanaendocrino@gmail.com); Diabetes and Hypertension Center of Ceará: Adriana Costa e Forti*, M.D. (adrianaforti@uol.com.br), *Cristina Figueiredo Sampaio Façanha*, M.D. (crisffacanha@hotmail.com); Federal University of Ceará: Renan Montenegro Junior*, M.D. (renanjr@ufc.br), *Ana Paula Montenegro*, M.D. (clinicarenanmontenegro@hotmail.com); Federal University of Sergipe: Naira Horta Melo*, M.D. (nhmelo@gmail.com), *Karla Freire Rezende*, M.D. (kfr@infonet.com.br); Federal University Hospital of Campina Grande, Paraíba: Alberto Ramos*, M.D. (ajsr@uol.com.br); Federal University Hospital of Pará: João Soares Felício*, M.D. (felicio.bel@terra.com.br), *Flavia Marques Santos*, M.D. (drafms@bol.com.br); Getúlio Vargas University Hospital of Amazonas, Adriano Jorge Hospital: Deborah Laredo Jezini*, M.D. (dljezini@hotmail.com).

## Abbreviations

(ADA): American Diabetes Association
(ADDQoL): Audit of Diabetes-Dependent Quality of Life
(ANOVA): analysis of variance
(BMI): body mass index
(DAFNE): Dose Adjustment for Normal Eating
(DCCT): Diabetes Control and Complications Trial
(DQOL): Diabetes Quality of Life
(EQ-5D): EuroQoL 5-Dimension
(FPG): fasting plasma glucose
(HbA1c): glycated haemoglobin
(HPLC): high-performance liquid chromatography
(HRQoL): health- related quality of life
(IBGE): Brazilian Institute of Geography and Statistics
(NBHCS): National Brazilian Health Care System
(QoL): quality of life
(SD): standard deviation
(SF-36): Short-Form 36
(Type 1 DM): Type 1 Diabetes Mellitus
(WHOQOL): World Health Organization Quality of Life
